# Improving aboveground biomass estimation in desert steppe using hyperspectral semantic segmentation

**DOI:** 10.3389/fpls.2026.1853466

**Published:** 2026-07-15

**Authors:** Xiaotian Sun, Haichao Wang, Zhiyong Pei, Kai Sun, Junyao Zhang, Yongbin Bao, Lina Shen, Wenxia Zhang, Guanzhi Liu

**Affiliations:** 1 College of Materials Science and Art Design, Inner Mongolia Agricultural University, Hohhot, China; 2 College of Mechanical and Electrical Engineering, Inner Mongolia Agricultural University, Hohhot, China; 3 School of Information Engineering, Ordos Institute of Technology, Ordos, China; 4 College of Grassland Science, Inner Mongolia Agricultural University, Hohhot, China

**Keywords:** aboveground biomass, feature selection, hyperspectral remote sensing, PLSR, semantic segmentation

## Abstract

**Introduction:**

Accurate estimation of aboveground biomass (AGB) in desert steppes is essential for evaluating ecosystem productivity, optimizing grazing management, and monitoring ecological degradation. However, AGB retrieval in these ecosystems remains challenging because sparse vegetation cover and exposed soil backgrounds often cause severe spectral mixing, thereby reducing the accuracy and robustness of biomass estimation models.

**Methods:**

We developed a hyperspectral AGB estimation framework that improves vegetation spectral purity through semantic segmentation and enhances model performance through spectral feature optimization. Using hyperspectral imagery from the desert steppe of Otog Banner, Ordos, Inner Mongolia, China, we first extracted grass vegetation to reduce background interference using U-Net, SegNet, DeepLabV3+, and random forest (RF). The extracted vegetation spectra were then optimized through spectral preprocessing, vegetation index integration, and feature selection, and subsequently subjected to partial least squares regression (PLSR) for AGB estimation. Model performance was evaluated using leave-one-out cross-validation (LOO-CV).

**Results:**

The U-Net had the best performance for vegetation extraction, with an overall accuracy (OA) of 0.923, effectively reducing interference from bare soil, shadows, and other non-vegetation backgrounds. The modeling combination (SNV+VI+CARS+PLSR) yielded optimal performance, with a coefficient of determination (R²) of 0.83, a root mean square error (RMSE) of 18.93 g/m², and a ratio of performance to deviation (RPD) of 2.39.

**Discussion:**

These findings demonstrate that hyperspectral semantic segmentation can effectively improve AGB estimation in desert steppes by enhancing vegetation spectral purity and reducing background contamination. The proposed framework provides an effective approach to biomass estimation in sparsely vegetated grassland ecosystems.

## 1 Introduction

Grassland ecosystems are important components of terrestrial ecosystems, covering approximately 26% of the global land surface and 41.7% of China’s total land area. They play important roles in climate regulation, biodiversity conservation, water retention, carbon cycling, windbreaks, and sand fixation (Kang et al., 2007). Among these ecosystems, the desert steppe represents a typical grassland type in arid and semi-arid regions. It serves as an essential foundation for maintaining regional ecological security and promoting the sustainable development of grassland-based animal husbandry. In recent years, grassland degradation and desertification in arid and semiarid regions have become increasingly severe, particularly in northern China, posing major challenges to the sustainable utilization of grassland resources and ecological management (Briske et al., 2015; He et al., 2015; Akiyama and Kawamura, 2007).

Aboveground biomass (AGB) is a key indicator of grassland vegetation status, productivity, and ecological function. Therefore, accurate estimation of AGB is of great importance for monitoring grassland degradation, regulating stocking rates, and assessing ecological restoration (Li et al., 2016). Previous studies on grassland AGB estimation have primarily relied on field-based surveys such as quadrat harvesting and biomass weighing. Although these methods provide relatively high accuracy, they are time-consuming, labor-intensive, offer limited spatial representativeness, and are difficult to apply to continuous monitoring at regional scales (Morais et al., 2021). With the development of remote sensing technology, grassland AGB estimation based on multi-platform data has attracted widespread attention, and related approaches have achieved promising results at the regional scale (Meng et al., 2020; Sun et al., 2025; Li et al., 2024). Among these approaches, hyperspectral remote sensing, which provides continuous narrowband spectral information, has demonstrated considerable potential for estimating AGB and related ecological parameters (Shen et al., 2008). Significant progress has been made in remote sensing studies across croplands (Wang et al., 2022; Yue et al., 2017), forests (Liu et al., 2024; Wu et al., 2025), and high-coverage grasslands (Ge et al., 2018; Zeng et al., 2019), demonstrating that remote sensing features exhibit stable relationships with AGB under conditions of high vegetation cover (or dense canopy closure). However, the desert steppe is characterized by sparse vegetation and extensive bare soil exposure, which results in a strong mixing of vegetation and background signals within individual pixels and substantially weakens the spectral response of the target vegetation. Relevant studies have shown (Elmore et al., 2000; Ren et al., 2018) that bare soil can substantially interfere with vegetation indices’ ability to characterize biomass, while endmember variability further increases the uncertainty of sub-pixel-scale inversion (Somers et al., 2011). Therefore, for low-coverage desert steppes, effectively separating the bare-soil background and improving the purity of vegetation spectral information have become critical to enhancing the accuracy of AGB inversion.

Deep learning–based semantic segmentation models, such as U-Net, have demonstrated excellent applicability in remote sensing object recognition (Ronneberger et al., 2015; Badrinarayanan et al., 2017; Chen et al., 2018; He et al., 2025; Yang et al., 2025), providing a technical foundation for the strategy of “first separating vegetation from the background and then retrieving biomass” in low-coverage desert steppe (Asner and Lobell, 2000). This approach not only significantly improves the purity of the target spectral information but also further confirms the potential of hyperspectral data for vegetation characterization in desert steppe ecosystems (Wang et al., 2025; Liu et al., 2023). However, even after pure vegetation information has been extracted, the inherent band redundancy and multicollinearity of hyperspectral data remain major bottlenecks, limiting inversion accuracy (Im and Jensen, 2008; Bioucas-Dias et al., 2013). In addition, with respect to modeling, compared with machine learning models such as random forest, partial least squares regression (PLSR) offers greater robustness and interpretability when handling high-dimensional features, making it more suitable for AGB estimation in desert steppe, where sample sizes are limited. Background interference is substantial (Song et al., 2025). Therefore, systematically comparing spectral preprocessing, vegetation index integration, and feature selection strategies under semantic segmentation masks while incorporating robust PLSR modeling is of great significance for revealing the synergistic relationship between vegetation extraction and feature optimization and for establishing an accurate AGB inversion framework.

Despite recent advances in hyperspectral remote sensing for grassland biomass estimation, accurately retrieving AGB from desert steppes remains challenging due to severe spectral mixing from sparse vegetation cover and complex background components, such as bare soil and litter. In addition, the high dimensionality and redundancy of hyperspectral data further limit model performance, particularly under small-sample conditions. Therefore, improving vegetation spectral purity and optimizing feature representation are critical steps to enhance AGB estimation accuracy in these ecosystems.

Therefore, this study aimed to (1) evaluate semantic segmentation models for grass vegetation extraction in desert steppe, (2) examine the effects of spectral preprocessing on vegetation spectral characteristics, (3) identify sensitive bands and vegetation indices related to AGB, (4) compare the performance of different feature combinations for AGB estimation, and (5) reveal the spatial distribution patterns of AGB in the study area. The results provide methodological support for accurate remote sensing retrieval of AGB in desert steppe ecosystems.

## 2 Materials and methods

**Figure 1:**
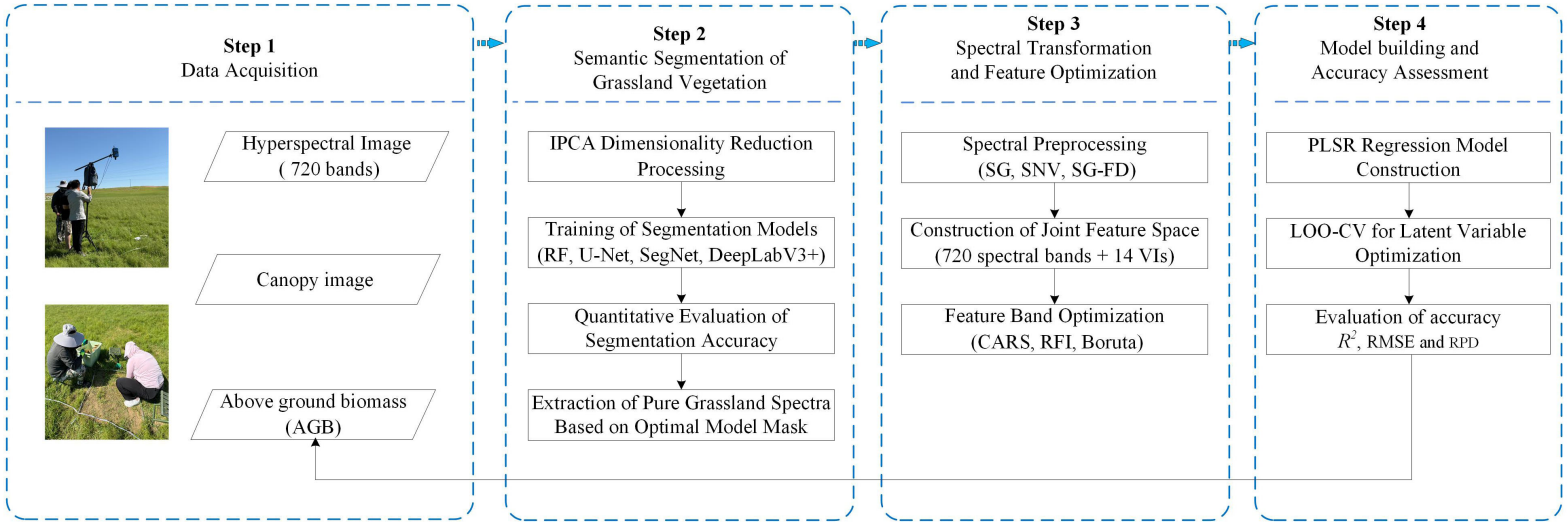
Experimental flow of this study.

This study was conducted in the desert steppe of Otog Banner, Ordos, Inner Mongolia, China, and proposes an AGB estimation method that integrates semantic segmentation with hyperspectral feature optimization. First, hyperspectral image data comprising 720 bands were acquired over the study area, and canopy images of the AGB were synchronously measured to construct a field-measured dataset for model training and validation. Second, semantic segmentation of the desert steppe vegetation was performed. The dimensionality of the hyperspectral data was first reduced using the IPCA method, and four segmentation models—RF, U-Net, SegNet, and DeepLabv3+—were trained separately. Their segmentation performance was quantitatively evaluated and compared, and the optimal model was selected to generate vegetation masks for extracting pure grassland spectra, thereby reducing interference from complex background components such as soil and litter. Subsequently, spectral transformation and feature optimization were performed. The extracted pure spectra were pre-processed using Savitzky–Golay smoothing (SG), standard normal variate (SNV), and Savitzky–Golay smoothing combined with Savitzky–Golay first-derivative transformation (SG-FD). A joint feature space was constructed by integrating the 720 original spectral bands with 14 vegetation indices (VIs). Feature selection was then performed using competitive adaptive reweighted sampling (CARS), random forest importance ranking (RFI), and Boruta, and the model was constructed and its accuracy assessed. Using the optimized feature variables as inputs and the measured AGB as the dependent variable, partial least squares regression (PLSR) was employed to develop an AGB estimation model for the desert steppe, and leave-one-out cross-validation (LOO-CV) was used to optimize the number of latent variables. Finally, the model performance was evaluated using the coefficient of determination (*R²*), root mean square error (RMSE), and ratio of performance to deviation (RPD) to identify the optimal technical combination for low-coverage grassland scenarios. The detailed experimental workflow is shown in **Figure 1**.

### 2.1 Study area

**Figure 2:**
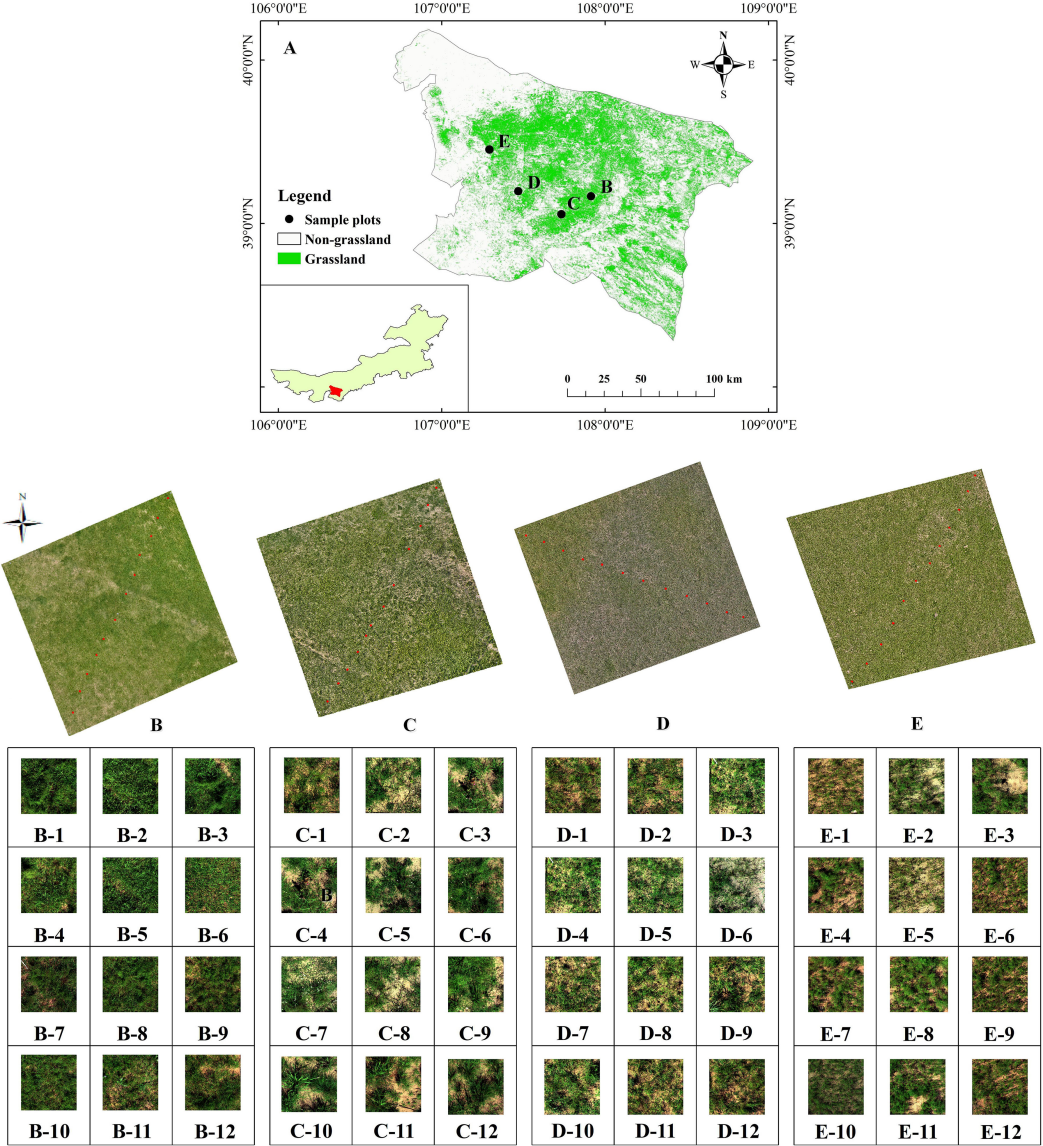
Location of the study area. Location and canopy image of sample plots. **(A)** Location of study area, **(B–D)** Canopy image of four plots.

The experimental site was located in Otog Banner, Ordos, Inner Mongolia Autonomous Region, China, between 106°41′–108°54′ E and 38°18′–40°11′ N (**Figure 2**). The grassland in the study area is classified as a desert steppe. The region has a typical temperate continental climate, with a mean annual precipitation of approximately 267 mm and a mean annual evaporation of approximately 2480 mm, which is nearly nine times the annual precipitation, indicating an overall arid climate. The growing season extends from April to September, during which precipitation reaches approximately 241.8 mm, accounting for 91.24% of the total annual precipitation. The long-term mean relative humidity across the banner was 48%. The vegetation community in the study area was dominated by *Stipa breviflora* as the constructive species, and *Artemisia scoparia* and *Chloris virgata* as the dominant species. The primary associated species were *Caragana stenophylla*, *Lespedeza bicolor*, *Polypogon fugax*, *Lespedeza potaninii*, *Allium mongolicum*, and *Eragrostis minor*.

### 2.2 Data acquisition

The field experiments were conducted from August 14 to 16, 2025. Based on long-term monitoring data from the Otog Banner Grassland Supervision Station and field reconnaissance results, four plots representing different degradation gradients were selected within the study area. All plots were characterized by calcic brown soils and exhibited low vegetation cover and high soil brightness, resulting in pronounced spectral mixing effects. In each plot, sampling transects were arranged along the diagonal direction, and 12–1 m × 1 m quadrats were established at equal intervals, yielding 48 quadrats.

#### 2.2.1 Spectral acquisition

Each quadrat was georeferenced using a centimeter-level GNSS-F200 receiver. Grassland hyperspectral data from the four study areas were acquired using an AHG101 portable hyperspectral imaging spectrometer. The instrument covers a spectral range of 400–1000 nm, with a spectral resolution of ≤3.5 nm, at least 1400 spectral channels, a dynamic range of ≥12 bit, and a lens better than F1.4/16 mm. Measurements were conducted under clear, cloud-free conditions with wind speeds below the Beaufort scale 3, and data were collected between 10:00 and 15:00 in Beijing to ensure stable illumination conditions. Before each acquisition, white reference calibration was performed using a standard reference panel to obtain the reflectance benchmarks. The spectrometer was mounted on a tripod, with the lens oriented downward at a height of approximately 2.5 m above the ground, providing a field of view approximately 0.6 m in diameter. Hyperspectral data were acquired using the built-in translational push-broom mode, in which the scanning speed was automatically matched to ensure distortion-free imaging. The integration time was set to 15–30 ms based on the ambient illumination conditions on the day of measurement, and image acquisition was manually triggered via software. During data collection, a standard reference panel was placed directly beneath the lens, occupying more than half of the field of view, to obtain digital number (DN) values. The panel was immediately removed, and hyperspectral data from the grassland canopy were collected. Each sampling point was measured three times, and the mean value was used as the representative spectrum for that point. After the hyperspectral data were collected for all sampling points, the raw DN data were stored for subsequent preprocessing.

#### 2.2.2 Ground data

1. Acquisition of canopy images.

To ensure complete imaging of each target ground quadrat (1 m × 1 m), the imaging height was set to at least 2.5 m, in accordance with the specifications of the AHG101 portable hyperspectral imaging spectrometer. Canopy images of all the quadrats were acquired and saved for subsequent processing.

2. AGB Measurement.

The vegetation within each quadrat was clipped at ground level, sorted by species, placed into labeled resealable bags, and weighed in the field to obtain fresh biomass. The samples were then transported to the laboratory, heated at 105 °C for 2 h to deactivate the enzymes, and oven-dried at 65 °C for more than 48 h until a constant weight was reached. The dry biomass was recorded, and the field-measured AGB included all aboveground plant material within each quadrat, comprising both green and non-photosynthetic vegetation (NPV).

### 2.3 Hyperspectral data pre-processing

To minimize the effects of illumination variability, instrument response characteristics, and environmental background on the spectral information, the raw data were preprocessed before analysis. This preprocessing workflow effectively reduced the influence of illumination fluctuations and sensor dark currents, thereby providing a reliable data foundation for the subsequent analysis of grassland spectral features and parameter retrieval. Preprocessing includes two main steps:

1. Radiance calibration.

Based on the manufacturer’s radiometric calibration file and the aperture settings used during data acquisition, the raw digital number (DN) images were calibrated to radiance using the AHG101 software, thereby generating radiance images and reducing the effects of sensor response nonuniformity.

2. Reflectance conversion.

During data acquisition, a standard reference panel was placed in each plot before imaging to ensure that it occupied more than half of the field of view. The region of interest (ROI) corresponding to the reference panel was extracted using ENVI, and the mean DN was calculated. Using the known absolute reflectance spectrum of the reference panel, the band-by-band reflectance calibration coefficients were derived and stored in a calibration coefficient file. Finally, these coefficients were applied to the radiance images using the Gain/Offset Correction tool in ENVI, completing the reflectance conversion and yielding hyperspectral reflectance data for the grassland canopy.

3. Spectral preprocessing.

To further reduce the spectral noise and baseline drift, the extracted reflectance spectra of the grassland canopy were preprocessed using three methods: Savitzky–Golay smoothing (SG), standard normal variate (SNV), and SG combined with first-derivative transformation (SG-FD). These preprocessing steps were applied before feature selection and subsequent AGB modeling.

### 2.4 Grassland vegetation segmentation

#### 2.4.1 Incremental principal component analysis

Incremental Principal Component Analysis (IPCA), an extension of Principal Component Analysis (PCA), was used to reduce the dimensionality of the hyperspectral data in this study. Given the large number of spectral bands and strong inter-band correlations in hyperspectral imagery, directly using the original 720-band data as input to semantic segmentation models would increase the computational complexity and reduce the training efficiency. The IPCA updates the principal components in a batchwise manner, reducing memory consumption and improving computational efficiency when processing large-scale pixel-level hyperspectral data.

The hyperspectral imagery used in this study contained 720 bands, spanning 396.8–1002.3 nm. Pixel-level spectra sampled from the 48 hyperspectral quadrats were used for the IPCA, and the first ten principal components (PC1–PC10) were retained. These ten components were then converted into 10-channel principal component images and used as inputs for subsequent semantic segmentation models. To clarify the retained components, their explained variance ratios, cumulative explained variances, peak loading bands, and peak loading coefficients are listed in **Table 1**. The peak loading band is defined as the wavelength corresponding to the maximum absolute loading coefficient of each principal component, and the peak loading coefficient refers to the original loading coefficient at that wavelength. The cumulative explained variance of PC1–PC10 reached 98.35%, indicating that the retained components preserved most of the spectral information from the original hyperspectral data while substantially reducing input dimensionality.

**Table 1:** Characteristics of the first ten principal components extracted by IPCA.

Principal component	Explained variance ratio (%)	Cumulative explained variance (%)	Peak loading band (nm)	Peak loading coefficient
PC1	81.3778	81.38	757.8	+0.0624
PC2	12.4912	93.87	684.7	+0.0766
PC3	2.8161	96.69	999.7	+0.0806
PC4	0.6562	97.34	757.8	+0.3131
PC5	0.4129	97.75	719.1	+0.0945
PC6	0.2478	98.00	696.7	+0.0870
PC7	0.1449	98.15	715.6	+0.0949
PC8	0.0913	98.24	759.6	+0.3214
PC9	0.0672	98.31	426.5	+0.0961
PC10	0.0459	98.35	938.0	+0.1656

Peak loading band refers to the wavelength with the maximum absolute loading coefficient for each principal component.

#### 2.4.2 Construction of the segmentation dataset

To construct a training dataset for semantic segmentation, this study adopted a semiautomatic annotation strategy that combines machine-based classification with manual correction. In ENVI, regions of interest (ROIs) were delineated on images of selected representative quadrats to obtain samples of three land-cover classes: grassland vegetation, bare soil, and shadow. Subsequently, the labeled ROI samples were used as training data to construct a random forest classifier, which was then applied to perform pixel-wise classification of the hyperspectral images from the 48 quadrats, generating the initial label maps. To address potential misclassifications along grassland boundaries and within mixed-pixel regions, a self-developed label-correction tool was employed to manually refine the initial classification results. The final labels were consolidated into two categories: grassland and non-grassland.

The first 10 principal component images and their corresponding labels were used as inputs, and the quadrat images were cropped into 256 × 256-pixel patches using a sliding-window approach, yielding 925 patches. During training, online data augmentation strategies, including random horizontal and vertical flipping and 90° rotation, were applied to expand the sample set. The dataset was then divided into training and validation sets in a 4:1 ratio.

#### 2.4.3 Build segmentation models

Vegetation cover in the desert steppe is relatively low, and substantial spectral mixing occurs between grassland vegetation and the bare-soil background. If the spectral information is extracted directly from the entire image, non-vegetation noise can be readily introduced. Therefore, it is necessary to first obtain grassland masks using segmentation models, and then perform subsequent spectral analysis and AGB retrieval based on the extracted grassland pixels. To this end, using the constructed 10-channel principal component images and binary grassland/non-grassland labels, this study employed four methods for grassland vegetation extraction: RF, U-Net, SegNet, and DeepLabv3 +.

1. Random Forest (RF).

RF is an ensemble-learning classification method based on a bagging strategy. It determines the class label of each pixel by constructing multiple decision trees and combining their outputs via majority voting (Breiman, 2001). RF offers several advantages, including fast training, strong robustness to noise, and low susceptibility to overfitting. Therefore, it has become one of the most widely used machine learning methods for remote sensing image classification. In this study, the reflectance values of all bands in the hyperspectral imagery were used as input features to develop a binary RF classifier for grassland and non-grassland classification.

Using the bootstrap method, random forest generates *B* sample subsets, denoted as *D_b_*, from the original training set *D*, as shown in **Equation 1**:

(1) $$D_{b}∼Bootstrap\;(D),\;b=1,\;2,\ldots ,B$$

For an input pixel *x*, the prediction of the *b-th* decision tree is expressed as follows, and the final classification result of the RF is determined by majority voting, as shown in **Equation 2**.

(2) $$\hat{y}(x)=\text{mode}\{{\hat{y}}_{1}(x),{\hat{y}}_{2}(x),\ldots ,{\hat{y}}_{B}(x)\}$$

At each split node, each decision tree randomly selects mmm candidate features from the full set of *M* features (typically $m\approx \sqrt{M}$) to enhance model diversity.

2. U-Net.

The U-Net is a classical semantic segmentation network with an encoder–decoder architecture. The encoder path consists of alternating convolutional and max-pooling layers, which progressively extract multiscale features while reducing spatial resolution. The decoder path gradually restores the spatial resolution through upsampling and convolution. The core innovation of U-Net lies in its skip connection mechanism, which concatenates shallow features from each encoder layer with deep features from the corresponding decoder layer, thereby recovering fine spatial details while preserving high-level semantic information and effectively improving boundary delineation accuracy (Ronneberger et al., 2015).

The core operation of U-Net is two-dimensional discrete convolution. For an input feature map *f* and convolution kernel *g*, the convolution operation is defined as **Equation 3**:

(3) $$\left(f*g)(i,j)=\sum_{\text{m}}\sum_{\text{n}}\;f(i-m,j-n)g(m,n\right)$$

For simplicity, each encoder stage was abstracted as a convolution, followed by max-pooling downsampling, as shown in **Equation 4**.

(4) $$x^{\left(l+1\right)}=\text{MaxPool}(\sigma (W^{\left(l\right)}*x^{\left(l\right)}+b^{\left(l\right)}))$$

where *σ* denotes the ReLU activation function, and *W*^(^*^l^*^)^ and *b*^(^*^l^*^)^ represent the convolution kernel and bias term of the *l*-th layer, respectively.

Through skip connections, the feature map $x_{\text{enc}}^{\left(k\right)}$ from the *k*-th encoder layer is concatenated along the channel dimension with the decoder feature map after upsampling, as shown in **Equation 5**.

(5) $$u^{\left(k\right)}=\text{UpSample}(x_{\text{dec}}^{\left(k\right)})⊕x_{\text{enc}}^{\left(k\right)}$$

where ⊕ denotes channel-wise concatenation.

In the output layer, a 1×1 convolution followed by a sigmoid activation function was used to map the feature map to the probabilities of grassland and non-grassland, as shown in **Equation 6**:

(6) $$\hat{y}=\sigma (W_{\text{o}ut}^{1\times 1}*u^{\left(0\;\right)}+b_{\text{o}ut})$$

3. SegNet.

SegNet is a deep-learning-based semantic segmentation network with an encoder–decoder architecture. Its encoder adopts the first 13 convolutional layers of VGG-16 for feature extraction. At the same time, the decoder restores features to their corresponding spatial locations during upsampling by reusing the indices of the maximum values recorded during the max-pooling operations in the encoding stage, thereby reducing information loss during upsampling (Badrinarayanan et al., 2017). Compared to U-Net, SegNet has fewer parameters and higher memory efficiency, making it suitable for large-scale remote sensing image segmentation tasks. During the encoding stage, SegNet recorded the positional indices of the maximum values in each pooling window, as shown in **Equation 7**.

(7) $$x_{\text{pool }},\;idx=\text{MaxPool}(x),\;idx\in {\text{Z}}^{\text{H}\times \text{W}}$$

During the decoding stage, the recorded indices are used for upsampling, and the feature values are restored to their original spatial positions, as shown in **Equation 8**.

(8) $$x_{\text{u}npool}=\text{M}axUnpool(x_{\text{u}p},idx)$$

This model used binary cross-entropy as the loss function. For a batch containing *N* pixels, the loss function is defined as **Equation 9**

(9) $$L=-\frac{1}{N}\sum_{i=1}^{N}[y_{i}\log{\hat{y}}_{i}+(1-y_{i})\log(1-{\hat{y}}_{i})]$$

4. DeepLabV3+.

DeepLabv3+ is an advanced semantic-segmentation network proposed by Google. It employs atrous convolution and an atrous spatial pyramid pooling (ASPP) module to enlarge the receptive field without reducing the spatial resolution, thereby effectively capturing multiscale contextual information. In addition, it incorporates an encoder–decoder architecture and depthwise separable convolution to improve the computational efficiency while maintaining the segmentation accuracy (Chen et al., 2018). DeepLabv3+ achieves state-of-the-art performance on multiple semantic segmentation benchmark datasets.

The atrous convolution enlarges the receptive field by inserting zero values between the elements of the convolution kernel. For the input *x*, the atrous convolution with dilation rate *r* is defined as **Equation 10**:

(10) $$\left(w{*}_{\text{atrous}}x)(i)=\sum_{k=0}^{K-1}w(k)\cdot x(i+r\cdot k\right)$$

where *K* denotes the kernel size, and *r* represents the atrous rate. When *r* = 1, the atrous convolution is reduced to a standard convolution.

The ASPP module applies multiple parallel atrous convolutions with different atrous rates together with image-level features, and concatenates the resulting feature maps along the channel dimension. For an output stride of 16, the ASPP module can be expressed as **Equation 11**.

(11) $$y_{ASPP}=\text{C}oncat(y_{1\times 1},y_{r-6},y_{r-12},y_{r-18},y_{\text{i}mage})$$

where *y_1×1_* denotes the 1×1 convolution branch (corresponding to *r* = 1); *y_r=6_*, *y _r=12_*and *y _r=18_* denote the 3×3 atrous convolution branches with atrous rates of 6, 12, and 18, respectively; *y_image_* denotes the image-level feature obtained by global average pooling. When the output stride was 8, the atrous rates were adjusted to 12, 24, and 36.

Depthwise separable convolution decomposes standard convolution into two steps, namely depthwise convolution and pointwise convolution, thereby significantly reducing the computational cost, as shown in **Equation 12**.

(12) Depthwise Separable Conv (x)=Pointwise Conv (Depthwise Conv (x))

Specifically, depthwise convolution performs convolution independently on each input channel, whereas pointwise convolution uses a 1×1 convolution to fuse cross-channel information.

### 2.5 Feature selection

Hyperspectral data are characterized by numerous spectral bands and a high degree of information redundancy. Directly using all bands for modeling may lead to the curse of dimensionality and overfitting. To identify sensitive bands that are closely associated with biomass from a high-dimensional feature space, three feature selection methods were employed: competitive adaptive reweighted sampling (CARS) (Li et al., 2009), random forest importance ranking (RFI) (Breiman, 2001), and Boruta (Kursa and Rudnicki, 2010).

### 2.6 Vegetation indices selection

To some extent, vegetation indices can characterize vegetation growth status and physiological traits. Based on the original reflectance spectra, 14 vegetation indices closely related to grassland biomass were selected for this study. The names and calculation formulae are listed in **Supplementary Table 1**.

### 2.7 Partial least squares regression

PLSR was employed to construct the AGB estimation model. PLSR is a widely used multivariate regression method for high-dimensional spectral data analyses (Li et al., 2024). By projecting the predictor matrix and response variables onto a low-dimensional latent space, PLSR can effectively address problems such as high dimensionality, information redundancy, and multicollinearity. In the present study, PLSR was used to construct an AGB estimation model.

The basic principle of PLSR is to extract latent variables from the predictor matrix *X* and response matrix *Y* such that the covariance between them is maximized. The decomposition of *X* and *Y* can be expressed as **Equations 13**, **14**.

(13) $$X=T_{h}\cdot P^{T}+E$$

(14) $$Y=U_{h}\cdot Q^{T}+F$$

where *T* and *U* are the score matrices of *X* and *Y*, respectively, *P* and *Q* are the corresponding loading matrices, and *E* and *F* are the residual matrices.

In this study, leave-one-out cross-validation (LOO-CV) was used to automatically optimize the number of latent variables with a search range of 1 to 15.

### 2.8 Analysis and evaluation

#### 2.8.1 Evaluation of segmentation models

The overall accuracy (OA), kappa coefficient, precision, recall, F1 score, and intersection over union (IoU) were used to quantitatively evaluate the segmentation results. In the confusion matrix, grassland was defined as the positive class, and non-grassland as the negative class. Accordingly, true positives (TP) denote the number of grassland pixels correctly classified as grassland, true negatives (TN) denote the number of non-grassland pixels correctly classified as non-grassland, false positives (FP) denote the number of non-grassland pixels incorrectly classified as grassland, and false negatives (FN) denote the number of grassland pixels incorrectly classified as non-grassland. N denotes the total number of pixels.

#### 2.8.2 Evaluation of regression models

AGB retrieval modeling was conducted using data from 48 quadrats to objectively evaluate the predictive performance of the regression models. Given the relatively small number of field-measured samples (*n* = 48), LOO-CV was employed in this study to assess the model accuracy. Compared to simple random splitting, LOO-CV ensures that each quadrat serves as an independent validation sample under small-sample conditions, thereby maximizing the use of available sample information, reducing the risk of overfitting on small datasets, and providing a more robust and reliable evaluation of retrieval accuracy (Chen and Guestrin, 2016). In addition, the quadrats were distributed along the diagonals of four sampling sites, with inter-site distances substantially larger than the quadrat size. This sampling design helps minimize potential spatial autocorrelation among samples and supports a more reliable assessment of model performance. The evaluation metrics were R², RMSE, and RPD. In general, RPD > 2.0 indicates good predictive ability, RPD > 2.5 indicates excellent predictive performance, and RPD < 1.4 indicates poor predictive ability (Rumelhart et al., 1986).

## 3 Results and analysis

### 3.1 Grassland vegetation extraction by semantic segmentation

The extraction accuracies of the four segmentation methods for grassland regions in the hyperspectral imagery are presented in **Table 2**. Among the three deep-learning models, U-Net achieved the best overall performance with OA, Kappa, Precision, Recall, F1, and IoU values of 92.3%, 84.4%, 91.7%, 90.5%, 91.1%, and 83.6%, respectively. DeepLabv3+ achieved a recall comparable to the U-Net’s but showed lower precision. SegNet yielded lower values than U-Net across all evaluation metrics.

**Table 2:** Accuracy comparison of grassland segmentation methods.

Method	OA	Kappa	Precision	Recall	F1	IoU
U-Net	0.923	0.844	0.917	0.905	0.911	0.836
DeepLabV3+	0.898	0.794	0.856	0.917	0.886	0.795
SegNet	0.883	0.762	0.845	0.891	0.868	0.766
RF	0.972	0.944	0.961	0.973	0.967	0.936

**Figure 3:**
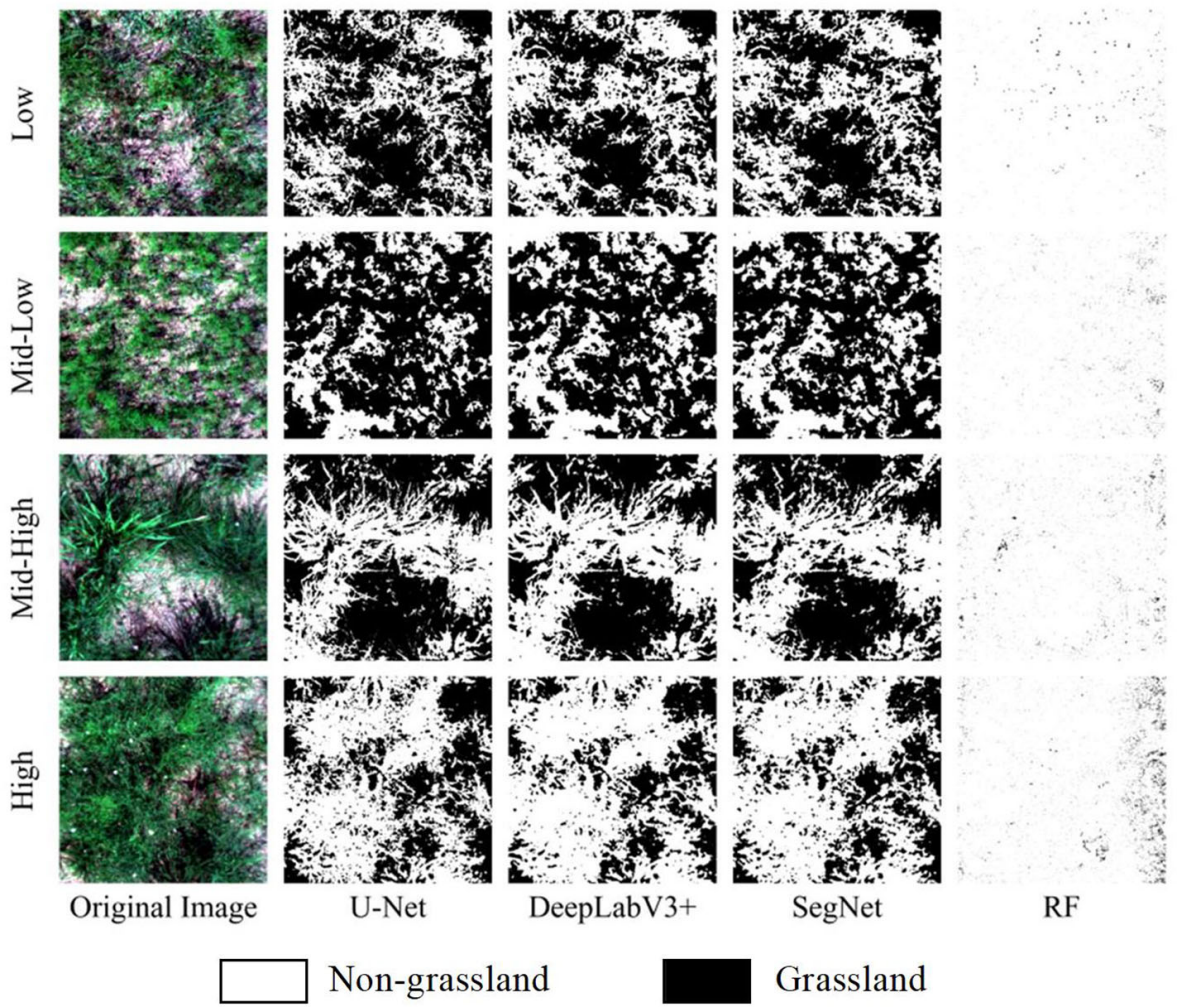
Comparison of grassland extraction results using different segmentation methods.

Although the RF reached relatively high pixel-level accuracy metrics, the segmentation results of the representative quadrats shown in **Figure 3** exhibited evident spatial overclassification, with many non-grassland regions misclassified as grassland. In contrast, the U-Net produced more continuous vegetation patches and clearer vegetation boundaries. Therefore, the U-Net was selected for subsequent vegetation mask generation and pure-vegetation spectral extraction.

### 3.2 Effects of spectral preprocessing on vegetation spectral responses

**Figure 4:**
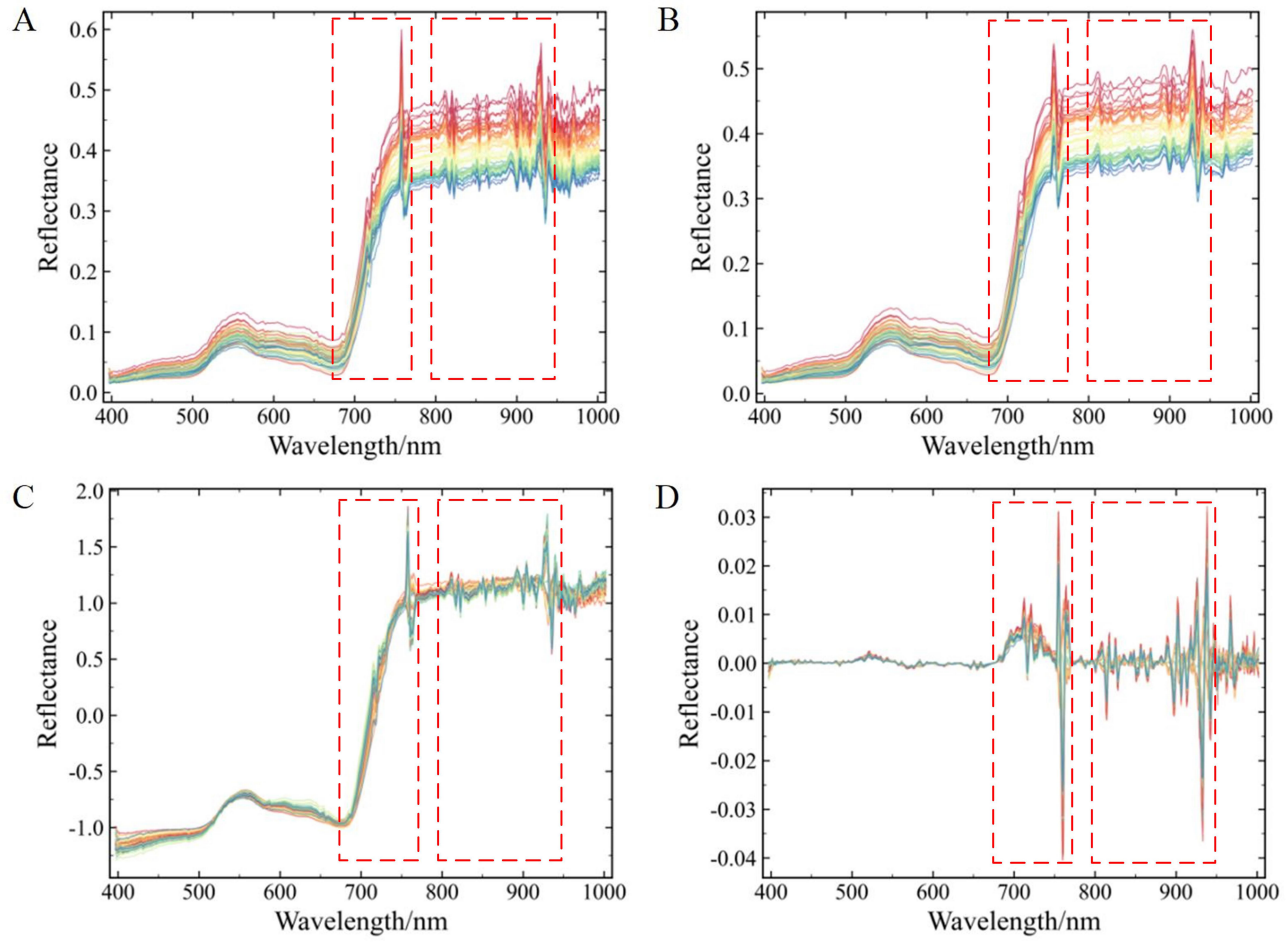
Effects of different spectral preprocessing methods on vegetation spectral responses. **(A)** Original spectra, **(B)** Savitzky–Golay smoothing (SG), **(C)** standard normal variate (SNV), and **(D)** Savitzky–Golay smoothing combined with first-derivative transformation (SG-FD).

The mean reflectance spectra of the grassland regions segmented by the U-Net model were analyzed to evaluate the effects of the different spectral preprocessing methods. A comparative analysis showed that SNV preprocessing most effectively corrected scattering effects while preserving informative spectral features, resulting in the highest AGB retrieval accuracy. Therefore, the SNV-preprocessed spectra were selected for all subsequent AGB modeling in the desert steppe (**Figure 4**).

### 3.3 Identification of biomass-sensitive spectral features

To fully exploit the biochemical information contained in the hyperspectral bands and the ability of vegetation indices to characterize canopy traits, 720 hyperspectral bands preprocessed by SNV were combined with 14 vegetation indices to construct a 734-dimensional joint feature space. The feature selection was performed using CARS, RFI, and Boruta.

#### 3.3.1 Feature selection by CARS

**Figure 5:**
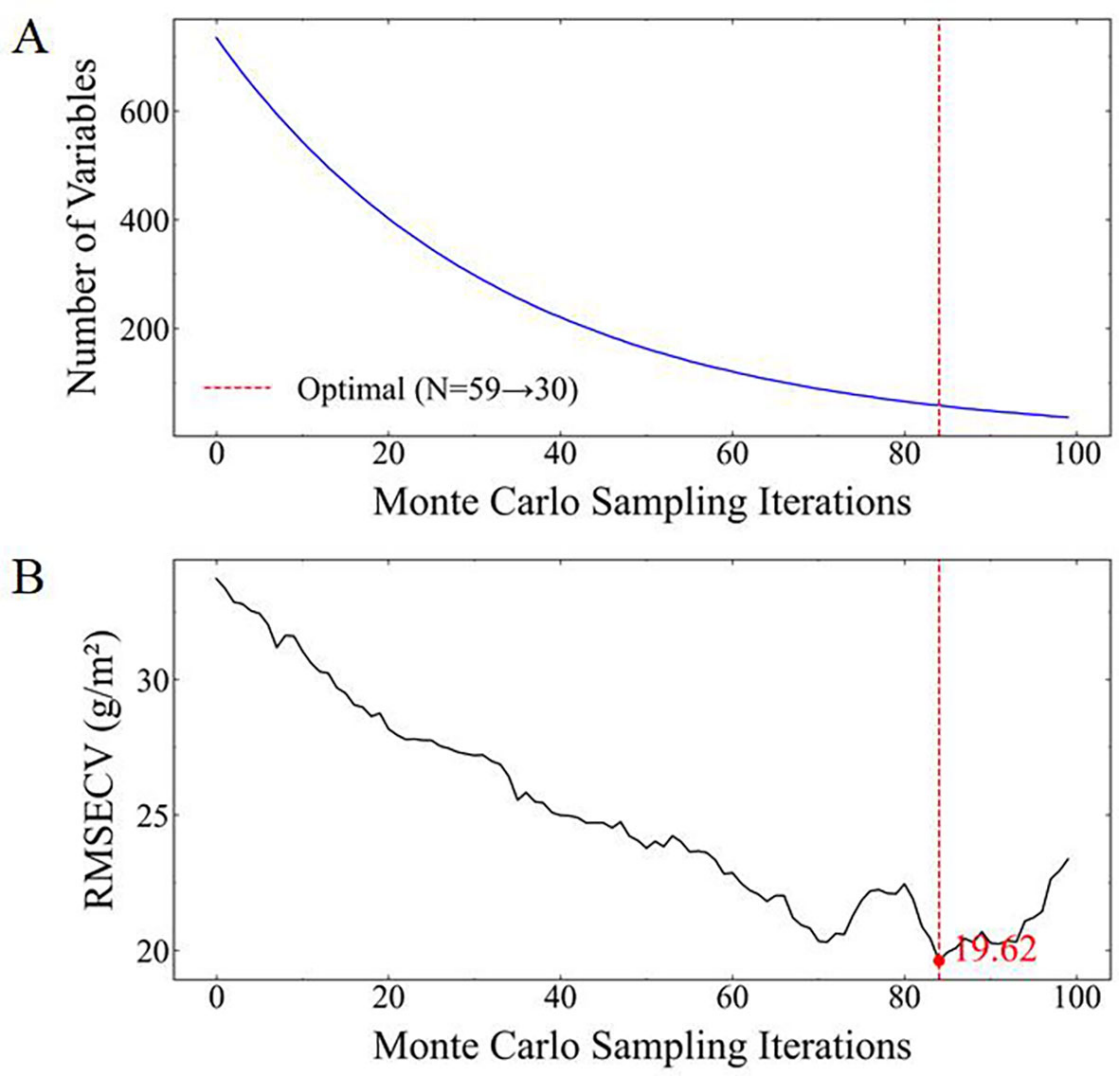
Feature selection results based on the CARS method. **(A)** Number of retained features during the 100 Monte Carlo sampling iterations, **(B)** RMSECV variation during the feature selection process, with the optimal feature subset identified at the 84th iteration.

The variable selection results of CARS during 100 Monte Carlo sampling iterations are shown in **Figure 5**. **Figures 5A, B** illustrate the variations in the number of retained features and RMSECV during the CARS selection process, respectively. Over 100 Monte Carlo iterations, the number of retained features generally decreased, whereas the RMSECV first decreased and then increased. At the 84th iteration, the RMSECV reached its minimum value of 19.62 g/m², and the corresponding feature combination was identified as the optimal subset. Ultimately, CARS selected 30 features from the initial 734 variables, including 29 spectral bands and 1 NDVI. These results indicate that CARS effectively reduces feature dimensionality while retaining a small number of representative vegetation index variables.

#### 3.3.2 Feature selection by RFI

**Figure 6:**
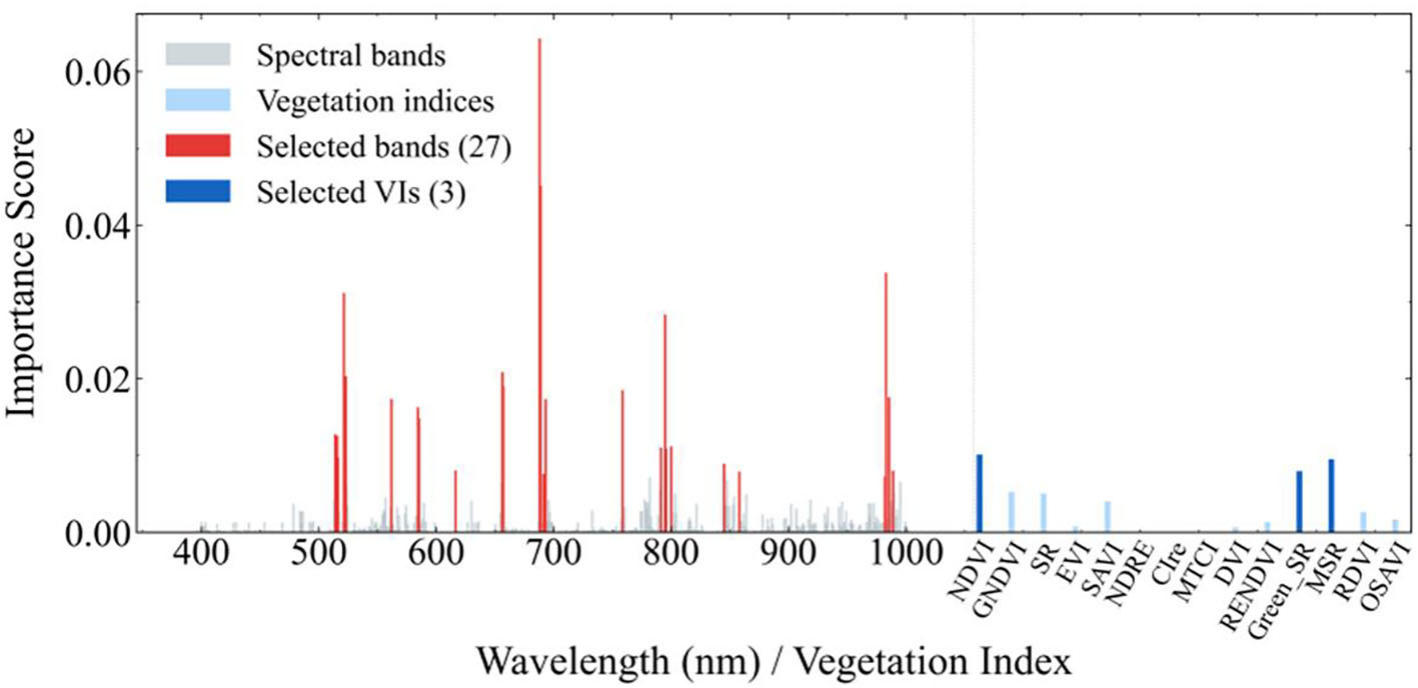
Feature importance ranking based on the random forest (RFI) method.

RFI evaluates feature importance by calculating the average reduction in impurity attributable to each feature during the construction of the decision trees. The feature-importance rankings derived from the RFI are shown in **Figure 6**. Based on the variable importance ranking, the top 30 variables were selected as the final retained features, including 27 spectral bands and three vegetation indices (NDVI, MSR, and GRVI). In terms of band distribution, the features selected by the RFI exhibited clear physical clustering, with particularly high importance weights in the 700–760 nm (red-edge region) and 780–900 nm (near-infrared plateau) regions, as indicated by the red bars. This finding highlights the high sensitivity of the red-edge bands to weak vegetation signals in desert steppes. Compared with CARS, the RFI method retained more vegetation index variables, indicating that under low-coverage conditions, nonlinearly combined vegetation indices contributed more to discrimination in the RF model than single spectral bands and were more effective at characterizing the spatial heterogeneity of biomass.

#### 3.3.3 Feature selection by Boruta

**Figure 7:**
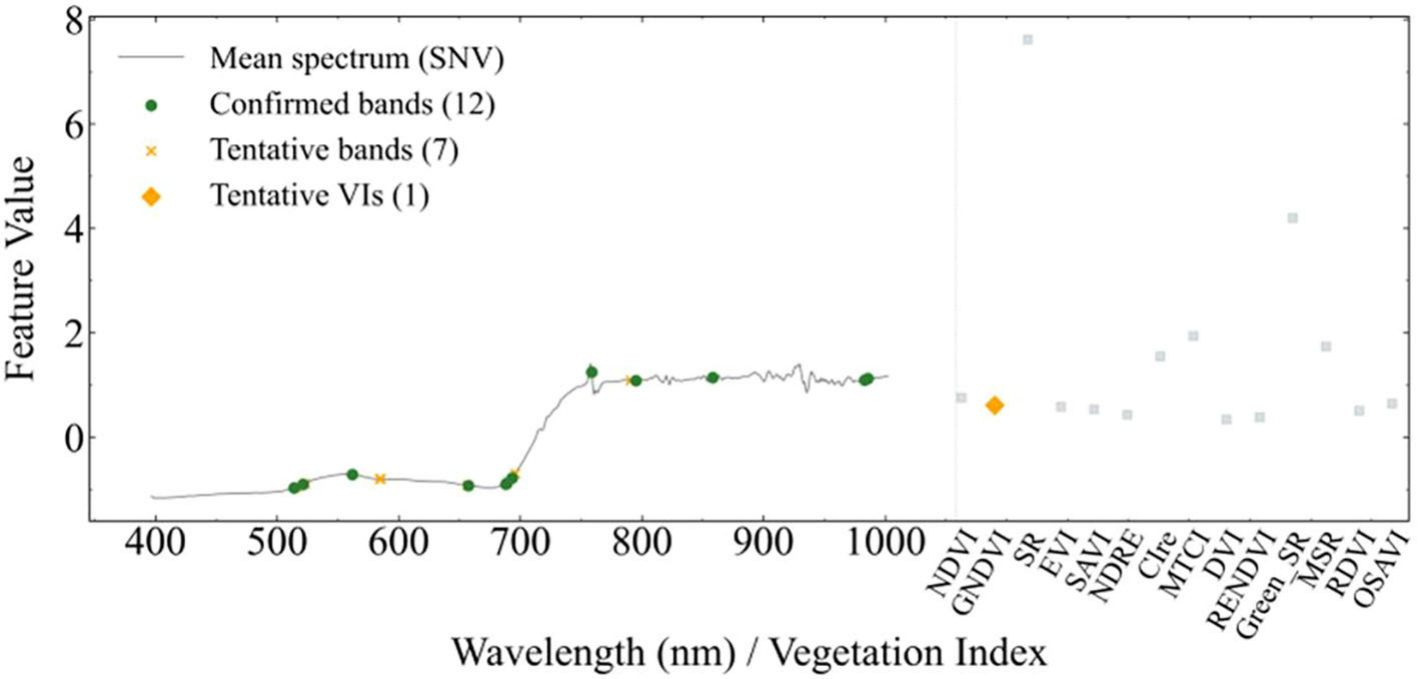
Feature selection results based on the Boruta algorithm.

Boruta identified all features relevant to the response variable by introducing shadow features. **Figure 7** shows the Boruta feature selection results for the 734-dimensional joint feature set. After iterative computation, Boruta confirmed 12 retained features, all of which were spectral bands, and identified eight tentative features, including seven spectral bands and one GNDVI. In subsequent modeling, the 12 confirmed spectral bands were used as the final input features for the Boruta method. Compared with CARS and RFI, Boruta retained the fewest variables.

Based on the above analysis, feature selection was conducted using CARS, RFI, and Boruta on 720 spectral bands and 14 vegetation indices. The results indicated that the red-edge (700–760 nm) and near-infrared (780–900 nm) regions were consistently retained across all methods, highlighting their strong contributions to AGB estimation. Vegetation indices such as NDVI, MSR, and GRVI were also repeatedly selected to collectively represent vegetation cover and canopy health. The red-edge region reflects chlorophyll content and photosynthetic activity; the near-infrared region captures leaf and canopy structural characteristics critical for identifying sparse vegetation; and the NDVI, MSR, and GRVI provide integrated information on vegetation coverage and health. Under the low-coverage conditions of the desert steppe, these key spectral features reliably capture biomass variability and are valuable for biomass estimation and ecological monitoring.

### 3.4 Development and validation of AGB estimation models

Under the two input scenarios of “spectra only” and “spectra + VIs”, three spectral preprocessing methods (SG, SNV, and SG-FD) were combined with three feature selection algorithms (CARS, RFI, and Boruta), resulting in 18 pre-processing–feature selection combinations. In addition, a PLSR model using only the VIs as input variables was constructed as a reference. Therefore, 19 models were developed, and their retrieval accuracies were evaluated using LOO-CV. The results are summarized in **Table 3**.

**Table 3:** Performance comparison of PLSR models under different spectral preprocessing methods and feature combinations for AGB estimation.

Modeling	Number of latent variables	Training set			Cross-validation		
		*R²*	RMSE	RPD	*R²*	RMSE	RPD
SNV+CARS+VI	7	0.92	12.61	3.59	0.83	18.93	2.39
SNV+CARS	8	0.89	15.06	3.01	0.77	21.66	2.09
SNV+Boruta+VI	10	0.77	21.79	2.08	0.53	30.95	1.46
SNV+Boruta	11	0.77	21.6	2.1	0.34	36.68	1.23
SNV+RFI+VI	5	0.74	23.09	1.96	0.34	36.93	1.23
SNV+RFI	9	0.78	21.02	2.15	0.30	37.98	1.19
SG+CARS+VI	4	0.67	26.09	1.74	0.75	22.87	1.98
SG+CARS	5	0.80	20.51	2.21	0.61	28.42	1.59
SG+Boruta+VI	1	0.58	29.52	1.53	0.53	31.03	1.46
SG+Boruta	1	0.45	33.49	1.35	0.31	37.55	1.21
SG+RFI+VI	1	0.59	28.99	1.56	0.55	30.47	1.49
SG+RFI	3	0.64	27.2	1.67	0.44	33.97	1.33
SG-FD+CARS+VI	8	0.89	15.03	3.01	0.55	30.52	1.48
SG-FD+CARS	7	0.87	16.19	2.8	0.51	31.68	1.43
SG-FD+Boruta+VI	1	0.60	28.64	1.58	0.47	33.03	1.37
SG-FD+Boruta	2	0.63	27.62	1.64	0.52	31.23	1.45
SG-FD+RFI+VI	1	0.57	29.79	1.52	0.38	35.8	1.27
SG-FD+RFI	4	0.70	24.77	1.83	0.40	34.95	1.3
VI	1	0.55	30.45	1.49	0.48	32.76	1.38

#### 3.4.1 Comparative analysis of pre-processing and feature selection combinations

**Figure 8:**
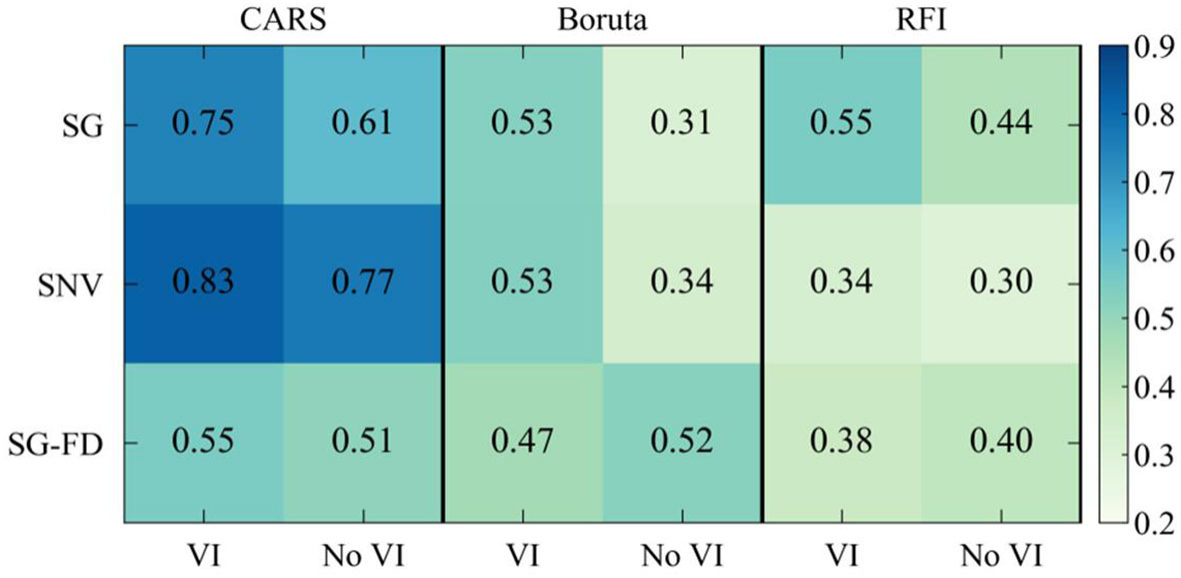
Comparison of PLSR model performance (*R²*) under different combinations of spectral preprocessing methods, feature selection strategies, and vegetation index integration.

**Figure 8** shows the performance differences among the PLSR models across combinations of preprocessing methods, feature selection strategies, and VI inclusion schemes, as indicated by the cross-validated coefficient of determination (*R²*). Overall, the preprocessing method had a significant impact on the PLSR model performance. Among the tested methods, SNV exhibited better adaptability across most technical combinations, and its corresponding models generally yielded higher *R²* values than those based on SG and SG-FD. Under the CARS-based feature selection conditions, the SNV-pre-processed model achieved the highest accuracy.

From a feature selection perspective, CARS generally outperformed Boruta and RFI. As shown in **Figure 8**, across all three preprocessing conditions, the CARS-based models consistently achieved relatively high *R²* values, with the SNV + CARS combination yielding the highest (*R²* = 0.83). By contrast, the models constructed using Boruta and RFI showed a lower overall accuracy under the same preprocessing conditions.

#### 3.4.2 Effects of vegetation index integration

The inclusion of vegetation indices (VIs) generally improved the retrieval performance of the PLSR models (**Figure 8**; **Table 3**). Across different combinations of preprocessing and feature selection, the models incorporating VIs achieved higher *R²* values than their spectra-only counterparts.

Among all combinations, the largest improvement was observed for the SNV + CARS combination after VI integration. Specifically, *R²* increased from 0.77 to 0.83, RMSE decreased from 21.66 g/m² to 18.93 g/m², and RPD increased from 2.09 to 2.39.

In contrast, the model constructed using only VIs as input variables achieved a relatively low accuracy (*R²* = 0.48), which was lower than that of the spectra–VI fusion models.

#### 3.4.3 Comparative analysis of representative PLSR model combinations

**Figure 9:**
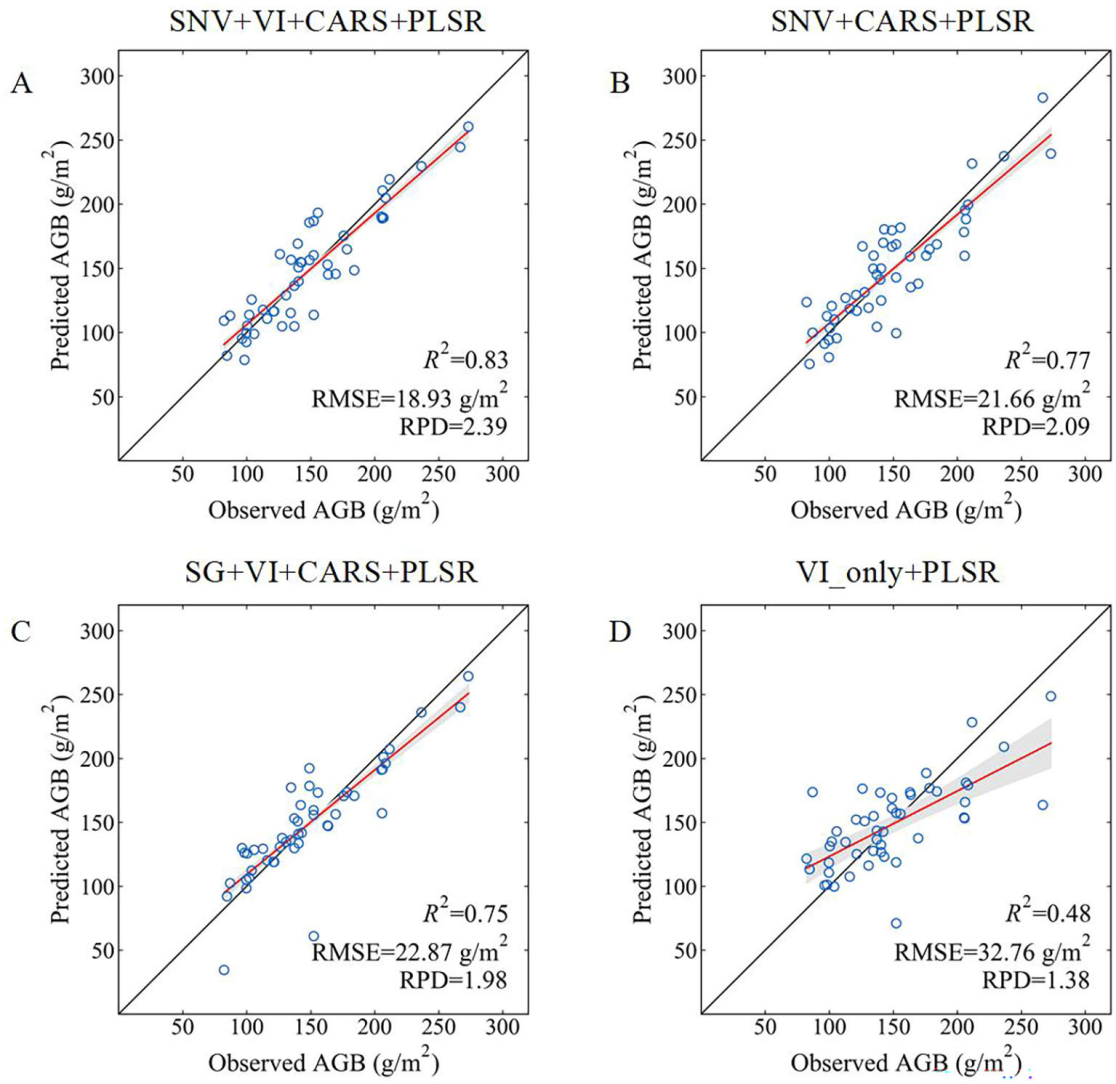
Scatterplots of observed versus predicted AGB for representative PLSR model combinations. **(A)** Scenario A: SNV+VI+CARS+PLSR; **(B)** Scenario B: SNV+CARS+PLSR; **(C)** Scenario C: SG+VI+CARS+PLSR; and **(D)** Scenario D: VI_only+PLSR. The 1:1 line indicates the agreement between observed and predicted values.

Scatter plots of the observed versus predicted values were generated for four representative PLSR model combinations (**Figure 9**). Overall, clear differences in the model-fitting performance were observed among the four scenarios. Scenario A (**Figure 9A**) exhibits the best performance, with scattered points tightly distributed around the 1:1 reference line. This model achieved the highest *R²* (0.83), lowest RMSE (18.93 g/m²), and RPD of 2.39.

The scatter distributions of Scenarios B–D were more dispersed. Scenario B (**Figure 9B**), which did not incorporate vegetation indices, still showed a relatively good fitting performance. However, scatter points in the high-biomass range were more dispersed than those in Scenario A. Scenario C (**Figure 9C**) maintained a relatively high fit but underestimated some high-value samples. Scenario D (**Figure 9D**), which used only VIs as input variables, showed the most dispersed scatter distribution, and the fitted regression line deviated further from the 1:1 reference line. This model also showed the lowest *R²* (0.48) and highest RMSE (32.76 g/m²).

#### 3.4.4 Ablation analysis of the proposed AGB estimation framework

To further evaluate the contributions of different components to the proposed AGB estimation framework, four ablation scenarios were designed (**Table 4**). Based on the preceding comparative analysis, SNV was selected as the spectral preprocessing method and CARS as the feature selection algorithm for the ablation experiments, as they achieved the best performance in PLSR modeling. Scenario S1 represented the baseline PLSR model constructed using the original spectra, without vegetation extraction, spectral preprocessing, vegetation index integration, or feature selection. Scenario S2 introduced U-Net-based vegetation extraction only, without spectral preprocessing, vegetation index integration, or feature selection, to verify the effect of removing non-vegetation background pixels. Scenario S3 incorporated SNV preprocessing, vegetation index integration, and CARS-based feature selection, but without vegetation extraction, to evaluate the independent contribution of spectral optimization. Scenario S4 represented the complete framework, incorporating all components: vegetation extraction, SNV preprocessing, vegetation index integration, and CARS feature selection.

**Table 4:** Ablation analysis of different components in the proposed AGB estimation framework.

Modeling	Training set			Cross-validation		
	*R²*	RMSE	RPD	*R²*	RMSE	RPD
S1	0.81	20.06	2.30	0.54	30.97	1.49
S2	0.84	18.17	2.52	0.58	29.43	1.56
S3	0.87	14.64	3.14	0.67	26.07	1.78
S4	0.92	12.61	3.59	0.83	18.93	2.39

The results showed that model performance gradually improved from S1 to S4. Compared with the baseline model S1, S2 achieved slightly higher accuracy, with training-set *R²* increasing from 0.81 to 0.84 and cross-validation *R²* increasing from 0.54 to 0.58. At the same time, RMSE decreased from 20.06 g/m² to 18.17 g/m² (training) and from 30.97 g/m² to 29.43 g/m² (cross-validation). Scenario S3 showed a more pronounced improvement: the training set *R²* reached 0.87, with an RMSE of 14.64 g/m² and an RPD of 3.14, whereas the cross-validation *R²* increased to 0.67 with an RMSE of 26.07 g/m² and an RPD of 1.78. The complete framework S4 achieved the best performance, with a training set *R²* of 0.92, RMSE of 12.61 g/m², RPD of 3.59; cross-validation *R²* of 0.83, RMSE of 18.93 g/m², and RPD of 2.39.

These results indicate that vegetation extraction, spectral preprocessing, vegetation index integration, and feature selection contributed to improvements in AGB retrieval accuracy. The best performance was obtained when all the components were integrated into a unified workflow.

### 3.5 Spatial distribution patterns of AGB in the desert steppe

**Figure 10:**
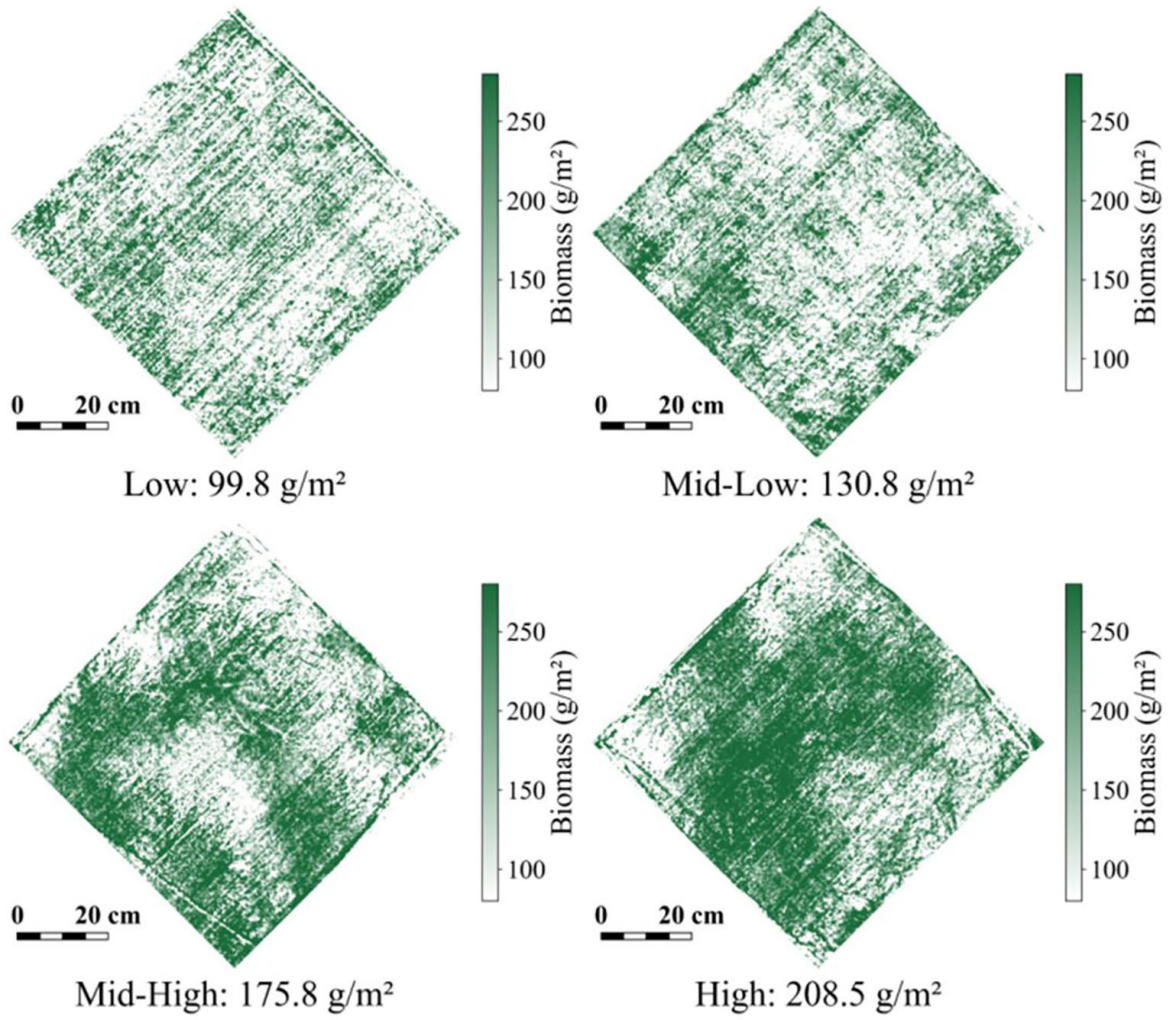
Spatial distribution maps of AGB retrieved using the optimal SNV+VI+CARS+PLSR model in four representative quadrats with different biomass gradients. The color gradient from nearly white or light green to dark green represents increasing AGB values.

Using the optimal SNV+VI+CARS+PLSR model, pixel-wise retrieval was performed based on ground-based hyperspectral data for representative quadrats with different biomass gradients in the study area, and spatial distribution maps of the grassland vegetation biomass were generated, as shown in **Figure 10**.

The retrieved spatial distribution patterns of biomass were generally consistent with ground observations. In each subfigure, the color gradually transitions from nearly white or light green to dark green, indicating an increase in the predicted biomass. White or very light areas primarily correspond to low-biomass vegetation or masked background pixels, whereas darker green areas represent higher AGB values.

A comparison between the retrieved mean biomass values and ground-measured values showed good consistency with the overall biomass gradient. In the low-biomass areas, the model captured the scattered distribution characteristics of sparse vegetation. In contrast, in the high-biomass areas, the retrieved mean values were close to the measured values, with a relative error of 0.19%.

## 4 Discussion

### 4.1 Semantic segmentation in desert steppe vegetation extraction and AGB estimation

Desert steppes are characterized by low vegetation cover and fragmented patches of vegetation, with extensive background components such as bare soil, litter, and shadows. These factors readily lead to spectral mixing between vegetation and non-vegetation signals, thereby increasing the uncertainty of AGB retrieval. Therefore, accurate vegetation extraction is a prerequisite for improving the reliability of biomass estimates. The results showed that U-Net achieved the highest overall accuracy (OA = 0.923) in the grassland semantic segmentation task, outperforming SegNet, DeepLabv3+, and RF. This indicates a stronger capability for vegetation discrimination under the complex background conditions of the desert steppe.

Compared with traditional pixel-wise classification methods, U-Net can simultaneously exploit multiscale semantic information and local spatial details, making it more effective in distinguishing fragmented vegetation patches from bare soil backgrounds. In contrast, RF relies mainly on pixel-wise spectral differences and is therefore more susceptible to spectral overlap between vegetation and bare soil under low-coverage conditions. Consequently, although RF achieved relatively high pixel-level accuracy metrics, its segmentation results exhibited evident spatial overclassification in the representative quadrats. Compared with RF, the U-Net preserved vegetation patch continuity and boundary information more effectively, thereby improving the spatial reliability of vegetation masks for subsequent spectral extraction.

This finding is consistent with the conclusions reported by He et al. (2025) for typical semi-arid grasslands. Using UAV imagery, He et al. compared the performance of PSPNet, DeepLabv3+, and U-Net in extracting photosynthetic and non-photosynthetic vegetation. They showed that deep learning–based semantic segmentation can effectively achieve fine-scale grassland vegetation identification under complex background conditions. Notably, PSPNet was identified as the optimal model in their study, whereas U-Net performed the best. This difference suggests that although deep-learning-based semantic segmentation is broadly effective in complex grassland scenarios, the performance of specific models may still depend on factors such as data source type, spatial resolution, classification targets, and sample structure, indicating a certain degree of scene dependency.

In addition, the introduction of semantic segmentation masks effectively excluded non-vegetation pixels such as soil, bare ground, and shadows, thereby improving the purity of the spectral information used in the subsequent hyperspectral analysis. This is consistent with the “segmentation before retrieval” strategy proposed by Asner and Lobell (2000), in which reducing background mixing effects provides a more reliable foundation for parameter retrieval. However, in areas with extremely low vegetation cover or weak spectral contrast, the segmentation results may still suffer from omission errors, indicating that the model performance is influenced by scene complexity and sample representativeness.

### 4.2 Effects of spectral preprocessing and feature selection on AGB retrieval

Following vegetation extraction, the extraction of informative variables from high-dimensional hyperspectral data is another critical factor influencing the accuracy of AGB retrieval. The results of this study showed that different spectral preprocessing and feature selection strategies had a pronounced impact on the model performance. Among all the tested combinations, SNV preprocessing combined with VI integration and CARS-based feature selection achieved the best retrieval performance. This indicates that under desert steppe conditions, relying solely on raw spectral information is insufficient to fully characterize vegetation biomass variation, whereas appropriate spectral correction and feature optimization can enhance biomass-related spectral responses.

From the perspective of spectral preprocessing, SNV outperforms SG smoothing and first-derivative transformation. This may be attributed to the high proportions of bare soil, senescent vegetation, and heterogeneous surface backgrounds in the desert steppe quadrats, making the original spectra more susceptible to baseline shifts and nonuniform scattering effects. By standardizing the spectra, the SNV can reduce multiplicative scattering interference from background mixing and surface-roughness differences, thereby improving spectral consistency across sampling points. In contrast, although the first-derivative transformation can enhance local spectral shape variations, it may also amplify spectral noise under low-coverage conditions, thereby limiting its contribution to model accuracy. Therefore, for desert steppe AGB retrieval, the role of preprocessing is mainly to reduce background interference and improve the separability of biomass-related spectral signals.

In terms of feature selection, CARS outperformed RFI and Boruta. The optimal CARS subset retained a limited number of informative variables, including 29 spectral bands and one vegetation index, and removed many redundant bands. This suggests that CARS can effectively compress feature dimensionality while retaining variables closely related to AGB variation, thereby reducing redundant information in PLSR modeling. In contrast, RFI and Boruta exhibited different variable-selection tendencies, with RFI retaining more vegetation indices and Boruta selecting fewer confirmed spectral bands. These differences indicate that the stability and biological relevance of the selected variables may vary among the feature selection algorithms.

The concentrations of the selected variables in the green, red-edge, and near-infrared regions suggest that these wavelength regions are important for characterizing AGB variation in desert steppes. The red-edge region is closely associated with chlorophyll content, canopy growth status, and leaf area. In contrast, the near-infrared region is more strongly related to vegetation cellular structure and canopy scattering characteristics. Compared with visible reflectance alone, these bands can provide more stable information for distinguishing differences in vegetation growth under low-coverage grassland conditions. In addition, the repeated retention of a small number of vegetation indices across different selection strategies indicated that index variables derived from band combinations can provide complementary information for AGB estimation, particularly for characterizing the canopy structure and reducing background interference.

The inclusion of vegetation indices further improved the retrieval performance of the PLSR models. In pairwise comparisons, models incorporating VIs generally yielded higher *R²* values than their spectra-only counterparts, indicating that VIs can supplement full-spectrum information for grassland biomass estimation. The improvement was most pronounced for the SNV+CARS combination after VI integration, with *R²* increasing from 0.77 to 0.83, RMSE decreasing from 21.66 g/m² to 18.93 g/m², and RPD increasing from 2.09 to 2.39. However, the model constructed using only VIs as input variables showed relatively low accuracy, indicating that VIs alone cannot replace the full spectral information for high-accuracy AGB retrieval from complex desert steppe backgrounds. These results indicate that VI integration can further improve AGB retrieval when combined with appropriate spectral pre-processing and feature selection.

### 4.3 Applicability of PLSR for AGB estimation in desert steppe

In this study, the SNV+VI+CARS+PLSR combination achieved the best estimation performance, with an *R²* of 0.83, RMSE of 18.93 g/m², and RPD of 2.39. This result indicates that PLSR can reliably estimate AGB in desert steppes under conditions of limited sample size and high collinearity among hyperspectral variables.

PLSR can address multicollinearity in high-dimensional data by extracting latent variables, thereby reducing model complexity, while retaining the main information related to the response variable. In this study, the number of field-measured samples was limited, whereas the hyperspectral data were characterized by high dimensionality and strong intervariable correlations. Under such “high-dimensional, small-sample” conditions, PLSR provides a balance between robustness and interpretability, which is consistent with previous studies on hyperspectral vegetation parameter estimation (Yue et al., 2017; Song et al., 2025). However, these results do not imply that PLSR outperforms all nonlinear models. Instead, they suggested that, given the current sample size, feature dimensionality, and study context, PLSR represents a relatively stable and interpretable modeling choice.

Moreover, the performance of PLSR in this study should be understood in relation to the preceding data processing steps. Semantic segmentation improved the purity of vegetation spectra, SNV reduced background-induced spectral variation, and CARS removed redundant variables before model construction. Therefore, the final retrieval accuracy reflects not only the modeling capability of PLSR but also the effectiveness of the overall workflow integrating vegetation extraction, spectral preprocessing, feature selection, and regression modeling.

Ablation analysis further confirmed that the improvement in retrieval accuracy was not attributable to a single processing step. Based on the preceding comparative analysis, SNV was selected as the spectral preprocessing method, and CARS as the feature selection algorithm, for the ablation experiments. Compared to the baseline model S1, vegetation extraction alone (S2) produced a moderate improvement. In contrast, spectral preprocessing, vegetation index integration, and CARS-based feature selection without vegetation extraction (S3) yielded a greater increase in model accuracy. The complete framework S4, which integrated all components, achieved the highest cross-validation performance, suggesting that synergy among vegetation extraction, spectral preprocessing, vegetation index integration, and feature selection is critical for improving AGB estimation in desert steppes.

### 4.4 Uncertainty and limitations

Although this study achieved promising results in terms of AGB estimation, it had several limitations. First, field quadrat data were mainly collected during a single growing season, specifically from July to August, and observations across different phenological stages were lacking. Therefore, the model’s applicability to other growth periods requires further investigation. Secondly, the sample size was relatively small. Although leave-one-out cross-validation was used to evaluate the model’s performance, its generalization across different regions and years still requires further testing.

In addition, segmentation uncertainty may also contribute to residual uncertainty in AGB retrieval, particularly in sparsely vegetated areas where omission errors reduce the representativeness of vegetation spectra and commission errors introduce soil background signals. Moreover, the robustness of the semantic segmentation model under different illumination conditions, acquisition times, and seasonal variations was not fully evaluated. Because changes in solar angle, shadow distribution, soil moisture, and vegetation phenology can affect spectral characteristics and image contrast, segmentation performance may vary across different environmental conditions. Future studies should incorporate multitemporal hyperspectral data and samples collected under diverse illumination conditions to train and validate models. Domain adaptation or transfer learning strategies could also be explored to improve the stability and generalizability of vegetation segmentation across different seasons and acquisition conditions.

Furthermore, desert steppe ecosystems exhibit strong spatial heterogeneity, with substantial differences in soil background, species composition, and vegetation cover among the regions. Therefore, the optimal feature combinations identified in this study may not be directly applicable to all grassland types. Future studies should integrate multitemporal hyperspectral data, larger-scale field samples, and a broader set of environmental covariates to improve further the model’s ability to characterize vegetation dynamics and enhance its cross-regional applicability. Moreover, although 14 vegetation indices were used in this study, additional band combinations and automated feature selection approaches could be explored to optimize AGB estimation further.

## 5 Conclusion

This study was conducted in the desert steppe of Otog Banner, Ordos, Inner Mongolia, China. An AGB estimation framework was proposed that integrates semantic segmentation with hyperspectral feature optimization to address the limitations of remote-sensing-based AGB estimation under low-coverage conditions caused by severe mixing between vegetation and background signals. The results showed that semantic segmentation effectively improved vegetation extraction under the complex background conditions of the desert steppe, with U-Net outperforming SegNet, DeepLabV3+, and random forest in distinguishing vegetation from non-vegetation components, such as bare soil and shadows, thereby providing more reliable spectral inputs for subsequent biomass estimation. Spectral preprocessing and feature selection further played critical roles in improving model performance, and standard normal variate (SNV) preprocessing and CARS-based feature selection showed the best applicability by reducing hyperspectral redundancy and multicollinearity while retaining informative variables closely related to vegetation growth. Among all tested combinations, the SNV+VI+CARS+PLSR framework achieved the best estimation performance, yielding an *R²* of 0.83, an RMSE of 18.93 g m^-^², and an RPD of 2.39. These findings indicate that improving spectral purity through semantic segmentation, together with appropriate preprocessing, feature selection, and PLSR modeling, can effectively enhance AGB estimation accuracy in desert steppes under conditions of limited sample size and strong collinearity among hyperspectral variables. Overall, this study provides a feasible and effective methodological framework for accurate remote sensing-based AGB estimation in low-coverage desert steppes. Future work should incorporate multi-temporal observations, multi-source remote sensing data, and cross-regional validation to improve the spatiotemporal applicability and generalization ability of the proposed framework.

## Data Availability

The original contributions presented in the study are included in the article/**Supplementary Material**. Further inquiries can be directed to the corresponding author.

## Supplementary material

Supplementary Table 1 Vegetation indices (VIs) used in this study.
