# Supplementary material for: Improving aboveground biomass estimation in desert steppe using hyperspectral semantic segmentation
Source: Front Plant Sci. 2026 Jul 15;17:1853466. doi: 10.3389/fpls.2026.1853466 (PMC13416253; doi:10.3389/fpls.2026.1853466)
Supplement: Supplementary Table 1 — Vegetation indices (VIs) used in this study. [file Table1.docx]

TABLE S1. Vegetation indices (VIs) used in this study.

| VI | Formula |
| --- | --- |
| Normalized Difference Vegetation Index（NDVI) | （R_800_ - R_670_）/（R_800_ + R_670_） |
| Green Normalized Difference Vegetation Index (GNDVI) | （R_800_ - R_550_）/（R_800_ + R_550_） |
| Ratio Vegetation Index (RVI) | R_800_ / R_670_ |
| Enhanced Vegetation Index (EVI) | 2.5（R_800_ - R_670_）/ R_800_ + 6R_670_ - 7.5R_470_ +1 |
| Soil-Adjusted Vegetation Index (SAVI) | 1.5（R_800_ - R_670_）/ R_800_ + R_670_ + 0.5 |
| Red-Edge Normalized Difference Vegetation Index (RENDVI) | （R_800_ - R_705_）/（R_800_ + R_705_） |
| Red-Edge Chlorophyll Index (CIred-edge) | R_800_ / R_705_ - 1 |
| MERIS Terrestrial Chlorophyll Index (MTCI) | （R_740_ - R_705_）/（R_705_ - R_670_） |
| Difference Vegetation Index (DVI) | R_800_ - R_670_ |
| Normalized Difference Red Edge Index (NDRE) | （R_740_ - R_705_）/（R_740_ + R_705_） |
| Green Ratio Vegetation Index (GRVI) | R_800_ / R_550_ |
| Modified Simple Ratio Index (MSR) | $\frac{R_{800}}{R_{670}}-1/\sqrt{\frac{R_{800}}{R_{670}}+1}$ |
| Renormalized Difference Vegetation Index (RDVI) | $R_{800}-R_{670}/\sqrt{R_{800}+R_{670}}$ |
| Optimized Soil-Adjusted Vegetation Index (OSAVI) | 1.16（R_800_ - R_670_）/ R_800_ + R_670_ + 0.16 |

Note: R_λ_ denotes reflectance at wavelength λ (nm). The selected wavelengths (e.g., 550, 670, 705, 740, and 800 nm) correspond to the green, red, red-edge, and near-infrared spectral regions commonly used in vegetation analysis.
